# Antepartum complications and perinatal mortality in rural Bangladesh

**DOI:** 10.1186/s12884-017-1264-1

**Published:** 2017-03-07

**Authors:** Rasheda Khanam, Saifuddin Ahmed, Andreea A. Creanga, Nazma Begum, Alain K. Koffi, Arif Mahmud, Heather Rosen, Abdullah H. Baqui, for the Projahnmo Study Group in Bangladesh

**Affiliations:** 1 International Center for Maternal and Newborn Health, Health Systems Program, Department of International Health, Johns Hopkins Bloomberg School of Public Health, Room – E8153, 615 North Wolfe Street, Baltimore, MD 21205 USA; 2 International Centre for Diarrhoeal Disease Research, Bangladesh (ICDDR,B), Dhaka, Bangladesh; 3 Department of Population and Family Health, Johns Hopkins Bloomberg School of Public Health, 615 N Wolfe Street, Baltimore, MD 21205 USA; 4 Washington DC, USA

**Keywords:** Antepartum complications, Perinatal deaths, Stillbirths, Early neonatal deaths, Antepartum hemorrhage, Probable infection, Pregnancy induced hypertension

## Abstract

**Background:**

Despite impressive improvements in maternal survival throughout the world, rates of antepartum complications remain high. These conditions also contribute to high rates of perinatal deaths, which include stillbirths and early neonatal deaths, but the extent is not well studied. This study examines patterns of antepartum complications and the risk of perinatal deaths associated with such complications in rural Bangladesh.

**Methods:**

We used data on self-reported antepartum complications during the last pregnancy and corresponding pregnancy outcomes from a household survey (*N* = 6,285 women) conducted in Sylhet district, Bangladesh in 2006. We created three binary outcome variables (stillbirths, early neonatal deaths, and perinatal deaths) and three binary exposure variables indicating antepartum complications, which were antepartum hemorrhage (APH), probable infection (PI), and probable pregnancy-induced hypertension (PIH). We then examined patterns of antepartum complications and calculated incidence rate ratios (IRR) to estimate the associated risks of perinatal mortality using Poisson regression analyses. We calculated population attributable fraction (PAF) for the three antepartum complications to estimate potential risk reductions of perinatal mortality associated them.

**Results:**

We identified 356 perinatal deaths (195 stillbirths and 161 early neonatal deaths). The highest risk of perinatal death was associated with APH (IRR = 3.5, 95% CI: 2.4–4.9 for perinatal deaths; IRR = 3.7, 95% CI 2.3–5.9 for stillbirths; IRR = 3.5, 95% CI 2.0–6.1 for early neonatal deaths). Pregnancy-induced hypertension was a significant risk factor for stillbirths (IRR = 1.8, 95% CI 1.3–2.5), while PI was a significant risk factor for early neonatal deaths (IRR = 1.5, 95% CI 1.1–2.2). Population attributable fraction of APH and PIH were 6.8% and 10.4% for perinatal mortality and 7.5% and 14.7% for stillbirths respectively. Population attributable fraction of early neonatal mortality due to APH was 6.2% and for PI was 7.8%.

**Conclusions:**

Identifying antepartum complications and ensuring access to adequate care for those complications are one of the key strategies in reducing perinatal mortality in settings where most deliveries occur at home.

## Background

Each year, an estimated 2.9 million babies die during the neonatal period [1] and another 2.6 million babies are stillborn [2] around the world. About three-fourths of the neonatal deaths occur within the first week of life [3, 4]. In developing countries, about two-thirds of stillbirths occur before the onset of labor [5] and one third during labor [2, 5]. Almost all perinatal deaths, which encompass neonatal deaths in the first week of life and stillbirths, occur in the developing world [2, 5]. The main causes of neonatal deaths include preterm related complications, intrapartum-related complications, and infections [1, 4, 6]. Impaired placental function is the main causal pathway proposed for stillbirths [2]. The primary known risk factors of stillbirths are presence of hypertensive disorders during pregnancy, obstetric complications, infections, placental dysfunction, and congenital defects [7–9]. Notably, many of these risk factors are also determinants of the main causes of neonatal deaths [4, 5].

Thus, a substantial proportion of perinatal deaths appear to have their origins in maternal complications during pregnancy. The most common complications are hemorrhage, hypertensive disorders of pregnancy, and infections [6, 10–13]. Antepartum hemorrhage beyond the first trimester is most often caused by placental abnormalities or incompetent cervix, and can result in stillbirth [6] and maternal death [10, 11]. The leading cause of hemorrhage during pregnancy is placental abruption which occurs in 1% of pregnancies and is associated with a perinatal case fatality of 10–30% [14]. Up-to 10% of women experience hypertensive disorders of pregnancy [15] and this condition can be associated with stillbirths, preterm birth, and neonatal or maternal deaths [15, 16]. Maternal infections such as malaria, syphilis, urinary tract infection, and bacterial vaginosis are also important causes of stillbirths [17–19] and important determinants of early neonatal deaths. Early onset neonatal infections may be acquired vertically during pregnancy or during delivery [20, 21].

In developing countries, where health systems are weak and care-seeking from health facilities is low, pregnant women often do not receive basic preventive and curative care when complications arise, or may delay care seeking [22] – such practices can result in stillbirths or neonatal deaths [23]. Understanding the associations between prevalent maternal complications during pregnancy and perinatal deaths is critical to guiding the development of strategies and programs to deliver maternal interventions of proven efficacy. The objective of this analysis is to examine patterns of maternal complications during pregnancy and associated risks of perinatal deaths in rural Bangladesh using household survey data of women who had a recent birth.

## Methods

### Study population

This study uses data from an endline household survey conducted for a cluster-randomized, controlled trial of a package of preventive and curative maternal-neonatal health interventions. The trial was conducted in rural Sylhet district of Bangladesh, located in the northeastern part of the country. Details of the study population and design were presented elsewhere [24]. Briefly, the trial was conducted between 2003 and 2006 in three rural sub-districts of Sylhet district. Twenty four unions each with about 20,000 population were randomized into three study arms: home-care, community-care, and comparison [24]. At baseline, neonatal mortality rate was 48 per 1,000 live births in the study area compared to 42 per 1,000 live births in the country as a whole [25], reflecting poorer socioeconomic status of the population as well as a poorer health systems compared to other parts of Bangladesh. Most health services are provided in the public sector including two community-based workers per population of 6,000–8,000; a first-level outpatient facility known as Union Health and Family Welfare Centers covering 20,000 population; and a sub-district hospital with both inpatient and outpatient services serving a population of about 200,000. The closest emergency obstetric-care facility is outside the study area at the Medical College Hospital in Sylhet City, which is about 75 km from the study site.

### Intervention

All married women of reproductive age (MWRA, age 15–49 years) were eligible to participate in the trial. The description of the intervention is presented in detail elsewhere [24]. Briefly, the home-care arm involved home visits by trained community health workers (CHWs) to promote a package of maternal and newborn care including home-based management of neonatal infection if referral to a hospital was not possible. CHWs, each serving about 700 married women of reproductive age (MWRA), identified pregnancies in their catchment area through routine 2-monthly household visits. Pregnant women were visited twice during pregnancy to promote birth and newborn care preparedness, including counseling and education on maternal and neonatal danger signs requiring treatment during pregnancy, delivery, and the postpartum period. CHWs also made 3 postnatal home visits on days 0, 2, and 6 of life to assess neonates and to identify and manage those with illnesses using an integrated management of childhood illness (IMCI) type clinical algorithms. The community-care arm received the same information through group education sessions [24] and the comparison arm received the standard care provided by outreach community-based workers and health facilities of the Ministry of Health and Family Welfare in Bangladesh.

### Data

The study included a baseline, three interim adequacy, and an endline household survey. The adequacy surveys were conducted in samples of households every 7–8 months between the baseline and endline surveys to assess the extent of intervention uptake. The endline survey was conducted in the entire study population and included a complete pregnancy history of all women of child bearing age. The pregnancy history involved enumeration of all pregnancies, pregnancy outcomes (miscarriage/abortion, stillbirths, and live births), survival status of all infants born alive on day 28, and age at death in days for those who died during the neonatal period. The endline survey also collected basic demographic and household wealth information from all women who had a pregnancy outcome in the three calendar years preceding the survey. To minimize recall error, data on knowledge and practices of maternal and new born care, selected key maternal complications, and intervention coverage data were collected from women who had a live or stillbirths between August and December 2005. To collect data on maternal complications during pregnancy, women were asked to report if they had: any vaginal bleeding but not spotting during antepartum period, fever, foul smelling vaginal discharge, convulsions, and swelling of feet or face during pregnancy [24, 26]. In this population many women were not literate. Data were collected by interviewers who made home visits and administered the questionnaire by interviewing women. The questionnaire was in local language.

### Measurement

The three outcomes of interest for this analysis are stillbirths, early neonatal deaths, and perinatal deaths. Stillbirth was defined as birth of a dead fetus after 7 months (≥28 weeks) of pregnancy. Early neonatal deaths are deaths that occurred within the first 7 days of life. Perinatal death is a composite of stillbirths and early neonatal deaths. We created three binary exposure variables indicating maternal complications during antepartum period: antepartum hemorrhage (APH; i.e., reports of any vaginal bleeding but not spotting during antepartum period); probable infection (i.e., having had fever and/or foul smelling vaginal discharge); and probable pregnancy-induced hypertension (PIH; i.e., having had convulsion and/or swelling of the feet or face). We characterized the last two complications as “probable” considering that these were self-reported and not clinically validated.

We examined the background characteristics of the women, including age (<25 years, 25–29 years, 30–34 years, and ≥35 years), educational attainment (no education, primary complete, and above primary) of women and their husbands, and household wealth status. A household wealth index score was constructed based on data on household assets using principal component analysis [27]. The households were ranked based on the wealth index score and categorized into quintiles. The lowest and highest quintiles were classified as poor and rich, respectively, relative to the three middle quintiles.

### Analysis

**Fig. 1:**
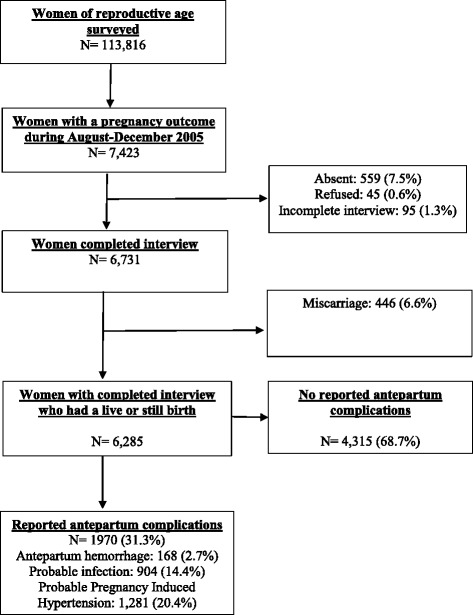
Analytic cohort of women

The unit of analysis was the woman. Of the 113,816 MWRA surveyed, 7,423 had a pregnancy outcome during the recall period and 6,731 completed the interview (Fig. 1). Three hundred sixty-six women had a miscarriage and thus, our analytic sample included 6,285 women who had given a birth during the recall period. Unadjusted rates and 95% confidence intervals (CI) of stillbirths, early neonatal deaths, and perinatal deaths were calculated by maternal complications during pregnancy. Multivariate Poisson regression analyses were conducted to estimate incidence rate ratios (IRR) of having adverse perinatal outcomes (stillbirths, early neonatal deaths, and perinatal deaths) for women with the three maternal complications of interest compared to those without these complications, adjusting for covariates. We also estimated the population attributable fraction (PAF) of having stillbirths, early neonatal deaths, and perinatal deaths for the antepartum complications from Poisson regression. PAF were estimated only when there was a significantly (p < 0.05) higher risk of an adverse outcome in the adjusted Poisson regression analyses. The PAF estimates the proportion of an adverse outcome that would be reduced at the population level following elimination of a maternal complication of interest, assuming that the relationship is causal [28, 29]. A *p*-value <0.05 was considered statistically significant. All analyses were adjusted for the complex survey design using Taylor’s linearization method. STATA 14 statistical software (Stata Corporation 2015, College Station, TX, USA) was used for all analyses.

## Results

Of the 6,285 women, about a third (31.3%) of the women reported a symptom consistent with at least one of the three antepartum complications examined in this study (Table 1). Approximately 6% of women reported a symptom consistent with two or more of the antepartum complications (data not shown). Socio-demographic characteristics of study participants are presented in Table 2. The mean (±SD) age of women was 28.1 (±6.1) years. Large proportions of women (41.2%) and their husbands (46.2%) did not attend school. Higher proportions of women with or without a maternal complication sought antenatal care and delivered in a facility or with a skilled attendant, but these proportions were low in both groups.

**Table 1:** Prevalence of self-reported maternal complications during pregnancy

Maternal complications	*n* = 6,285	%
Antepartum hemorrhage	168	2.7
Probable infection^a^	904	14.4
High fever	681	10.8
Foul smelling discharge	310	4.9
Probable pregnancy-induced hypertension^b^	1,281	20.4
Convulsion	161	2.6
Swelling of feet or face	1,169	18.6
Any of three complications	1,970	31.3

^a^Defined as women who reported having either high fever or foul smelling discharge during pregnancy

^b^Defined as women who reported having either convulsions or swelling of feet or face during pregnancy

**Table 2:** Socio-demographic and delivery-related characteristics of the study population

Characteristics	Total *N* = 6,285	None of three complications *N* = 4,315	Any of three complications *N* = 1,970	*p* value
	n (%)			
Women’s age				<0.001
< 25 years	1,763 (28.1)	1,242 (28.8)	521 (26.5)	
25–29 years	2,010 (32.0)	1,414 (32.8)	596 (30.3)	
30–34 years	1,339 (21.3)	918 (21.3)	421 (21.4)	
≥ 35 years	1,173 (18.6)	741 (17.2)	432 (21.9)	
Parity				
1–2	2,163 (34.4)	1,491 (34.6)	672 (34.1)	<0.010
3–4	1,936 (30.8)	1,382 (32.0)	554 (28.1)	
5–6	1,200 (19.1)	779 (18.1)	421 (21.4)	
7+	986 (15.7)	663 (15.4)	323 (16.4)	
Women’s education				0.948
No education	2,591 (41.2)	1,773 (41.1)	818 (41.5)	
Primary complete	1,926 (30.7)	1,326 (30.7)	600 (30.5)	
Above primary	1,768 (28.1)	1,216 (28.2)	552 (28.0)	
Husbands’ education				
No education	2,904 (46.2)	2,004 (46.4)	900 (45.7)	0.760
Primary complete	2,093 (33.3)	1,437 (33.3)	656 (33.3)	
Above primary	1,288 (20.5)	874 (20.3)	414 (21.0)	
Household asset index				
1 (Lowest)	1,102 (17.5)	787 (18.2)	315 (16.0)	<0.010
2	1,190 (18.9)	843 (19.5)	347 (17.6)	
3 (Middle)	1,252 (19.9)	866 (20.1)	386 (19.6)	
4	1,334 (21.2)	908 (21.0)	426 (21.6)	
5 (Highest)	1,407 (22.4)	911 (21.1)	496 (25.2)	
ANC visits				
0	2,142 (34.1)	1,673 (38.8)	469 (23.8)	<0.001
1–3	3,253 (51.8)	2,172 (50.3)	1,081 (54.9)	
4+	890 (14.2)	470 (10.9)	420 (21.3)	
Place of delivery				
Home delivery	5,396 (85.9)	3,825 (88.6)	1,571 (79.8)	<0.001
FWC/UHC	417 (6.6)	239 (5.5)	178 (9.0)	
Hospital/Clinic	431 (6.9)	227 (5.3)	204 (10.4)	
Other	41 (0.6)	24 (0.6)	17 (0.9)	
Birth attendant				
Untrained	3,767 (60.0)	2,675 (62.0)	1,092 (55.4)	<0.001
TBA	1,573 (25.0)	1,111 (25.8)	462 (23.5)	
Skilled birth attendant^a^	945 (15.0)	529 (12.3)	416 (21.1)	

*P*-value based on chi-square tests comparing women with and without complications

*ANC*, antenatal care, *FWC*, family welfare center, *UHC*, upazila health complex, *TBA,* traditional birth attendant

^a^Includes home and facility births assisted by skilled birth attendants (i.e., doctors, nurses, midwives)

Unadjusted rates and 95% CIs of adverse perinatal outcomes are presented in Table 3. The rate of stillbirths was 31 per 1,000 births (95% CI 27.0–35.6), rate of early neonatal deaths was 26 per 1,000 live births (95% CI 22.7–30.8), and the overall perinatal deaths rate was 57 per 1,000 births (95% CI 51.2–62.6). Notably, the rate of perinatal deaths was almost five times higher for women with APH (202.4 per 1,000 births, 95% CI 148.3–269.9) compared to those who had none of the three maternal complications (44.5 per 1,000 births, 95% CI 38.7–51.1).

**Table 3:** Rates of stillbirths, early neonatal deaths, and perinatal deaths by presence of maternal complications during pregnancy

Complications	Stillbirths^a^	Early neonatal deaths^b^	Perinatal deaths^a^
	n (rate, 95% CI)		
APH	20 (119.0, 78.1–177.3)	16 (94.6, 56.8–153.4)	34 (202.4, 148.3–269.9)
Probable infection	35 (38.7, 27.9–53.5)	37 (41.4, 30.0–56.9)	71 (78.5, 62.7–98.0)
Probable PIH	64 (50.0, 39.3–63.3)	43 (33.7, 24.9–45.4)	105 (82.0, 68.1–98.3)
Any of three complications	94 (47.7, 39.1–58.1)	70 (37.3, 29.6–46.9)	164 (83.2, 71.8–96.3)
None of three complications	101 (23.4, 19.3–28.4)	93 (21.6, 17.6–26.4)	192 (44.5, 38.7–51.1)
Overall rate	195 (31.0, 27.0–35.6)	161 (26.4, 22.7–30.8)	356 (56.6, 51.2–62.6)

*APH*, antepartum hemorrhage, *PIH*, pregnancy induced hypertension

^a^Rate per 1,000 births

^b^rate per 1,000 live births

Table 4 shows adjusted incidence rate ratios (IRR), corresponding 95% CIs, and PAF for adverse perinatal outcomes associated with each of the three maternal complications. Women who had experienced APH had a significantly higher likelihood of their fetus or neonate having an adverse outcome, either perinatal death (IRR = 3.5, 95% CI 2.4–4.9), stillbirth (IRR = 3.7, 95% CI 2.3–5.9), or early neonatal death (IRR = 3.5, 95% CI: 2.0–6.1) compared to those who did not experience APH. Probable PIH was a significant risk factor for perinatal deaths (IRR = 1.5, 95% CI: 1.2–1.9) primarily due to higher likelihood of having a stillbirth (IRR = 1.8, 95% CI 1.3–2.5). Probable maternal infection was a significant risk factor for early neonatal deaths (IRR = 1.5, 95% CI: 1.1–2.2).

**Table 4:** Incidence rate ratios and population attributable fractions of stillbirths, early neonatal deaths, and perinatal deaths during pregnancy by maternal complications

Complications	Stillbirths		Early neonatal deaths		Perinatal deaths	
	Adjusted IRR (95% CI)	PAF % (95% CI)	Adjusted IRR (95% CI)	PAF % (95% CI)	Adjusted IRR, 95% CI	PAF, % 95% CI
APH	3.7 (2.3–5.9)	7.5 (3.6–11.2)	3.5 (2.0–6.1)	6.2 (2.1–10.2)	3.5 (2.4–4.9)	6.8 (4.2–9.3)
Probable infection	1.1 (0.7–1.6)	–	1.5 (1.1–2.2)	7.8 (0.1–14.9)	1.3 (1.0–1.7)	–
Probable PIH	1.8 (1.3–2.5)	14.7 (6.4–22.3)	1.3 (0.9–1.8)	–	1.5 (1.2–1.9)	10.4 (4.7–15.7)
Total		20.7 (12.3–28.2)		13.1 (5.3–20.2)		16.2 (10.4–21.5)

All three Poisson regression models are adjusted for maternal age, parity, and household wealth

*APH*, antepartum hemorrhage, *PIH*, pregnancy induced hypertension, *PAFs* were estimated only for maternal complications found to be significantly associated at *p* < 0.05 with specific outcomes in Poisson regression analyses, *IRR*, incidence rate ratio, *PAF*, population attributable fraction

The PAF of perinatal mortality was about 6.8% for APH and 10.4% for probable PIH, lower than corresponding stillbirth PAF of 7.5% for APH and 14.7% for probable PIH. PAF of early neonatal mortality risk was 6.2% for APH and about 7.8% for probable infection. The PAF of perinatal deaths, stillbirths and early neonatal deaths were 16.2, 20.7 and 13.1% for all three complications combined.

## Discussion

We studied prevalence of three self-reported antepartum complications in rural Bangladeshi women and examined the risks of perinatal mortality associated with them. The burden of at least one of the three complications examined in this study i.e., APH, probable infection, and probable PIH, based on self-reported symptoms, was high (31.3%) and these antepartum complications were significantly associated with perinatal deaths. More specifically, APH was associated with increased risk of both stillbirths and early neonatal deaths, probable infection was associated with increased risk of early neonatal deaths, and probable PIH with an increased risk of having a stillbirths. Together with the high perinatal mortality and high PAF estimated for APH and PIH, these findings highlight the importance of promoting recognition of, care-seeking for, and management of antepartum complications.

Our findings show similar higher risks of perinatal deaths associated with APH and probable PIH as reported earlier from Bangladesh [12] and elsewhere [11, 16]. Mamun et al. [12] examined the patterns of maternal complications during different stages of gestation and their association with perinatal deaths using data from a community based clinical trial. After adjusting for potential confounders, the study documented that perinatal mortality was 2.7 times higher (95% CI 1.5–4.9) among women who had hypertension during pregnancy and 5.0 times higher (95% CI 2.3–10.8) among those who had experienced antepartum hemorrhage [12]. Two facility based studies, one conducted in South Africa and the other in Northwest Ethiopia demonstrated higher risk of stillbirths with hypertension and antepartum hemorrhage [11, 16]. Allanson et al. reported [16] that APH was significantly more common in women who had stillbirths (16.3%) compared to the women who had early neonatal deaths (7.4%). Similarly more women who had stillbirths had reported hypertension (23.6%) compared to women who had early neonatal deaths (8.1%) [16]. Adane et al. conducted a study [11] in Northwest Ethiopia and reported that stillbirths were significantly associated with both APH (AOR 8.4, 95% CI 1.3–55.3) and hypertension (AOR 9.5, 95% CI 2.1–44.3) [11]. The prevalence of maternal complications in both South African and Ethiopian studies were higher than our study likely due to differences in setting and populations studied.

The World Health Organization conducted a large facility based cross-sectional survey on maternal and newborn health in 29 countries in Asia, Africa, Latin America, and the Middle East and reported on perinatal indicators and risks of perinatal deaths in the presence of hemorrhage, infections, hypertensive disorders and other maternal complications [7]. Since this was a facility based survey, more detailed information on maternal complications were available and therefore, the results are not directly comparable to our study findings. The study reported that vast majority of perinatal deaths in participating facilities occurred in the presence of a maternal complication and concluded that understanding these relationships are critical in settings where maternal complications are often common, under-diagnosed, and/or under-treated and where perinatal mortality rates are high [7].

Our study has several limitations. We recognize that the data is somewhat old, however, health indicators in our study division (Sylhet) of Bangladesh did not change much in the last decade suggesting that our data and findings are still relevant. According to Bangladesh demographic and health survey (BDHS) 2007, perinatal mortality rate (PMR) in our study area was 69 per 1000 live births. PMR was 63 per 1000 live births according to BDHS 2014. Early neonatal mortality rate did not change (22/1,000 live birth in BDHS 2007 vs 23/1,000 live birth in BDHS 2014) [30, 31]. The cross sectional study design has its inherent limitations to imply causality. However, the strength of the study is population-based data and large sample size. The data were collected retrospectively, however we feel that recall bias was not a major issue as the recall period was short. Nonetheless, it is possible that mothers who experienced perinatal deaths were more likely to recall antepartum complications compared to mothers who did not experience a complication (selective recall).

Our data on antepartum complications are self-reported by the mothers and this raises concern regarding potential misclassification of the reported complications. Our prevalence estimates for probable PIH and probable infection were higher than those reported in the literature based on clinical examinations [32]. Clinical measures of complications are difficult to obtain outside health facilities, and our survey was conducted in a population where most women delivered at home and only few sought care from health facilities for antepartum complications. We acknowledge that the reported antepartum complications in our study was likely to be an over-estimate compared to the rate from other studies conducted in hospital/health care settings. However, we restricted our assessment of antepartum complications to only three conditions that have specific clinical signs that are easily recognizable by women and make these maternal conditions highly probable. We had information on a limited number of potential confounders of the relationships of interest in this study, and this may have led to an overestimation of the risk of perinatal deaths associated with antepartum complications and corresponding PAF. For example, we did not examine key maternal conditions known to increase the risk of stillbirths and early neonatal deaths including malnutrition, history of smoking, and diabetes. Misclassification of early neonatal deaths as stillbirths may be of concern as babies who die immediately after birth might have been reported as stillbirths irrespective of the place of delivery. To alleviate this problem, we measured the risks of the three maternal complications of interest and PAF for the composite indicator of perinatal mortality in addition to examining stillbirths and early neonatal deaths separately.

## Conclusions

The burden of antepartum complications in our population was high and since these conditions were shown to significantly increase the risk of perinatal mortality, their prevention and management in a timely manner are crucial to reducing perinatal mortality in Bangladesh and in similar settings. The utilization of antenatal care and facility delivery rate, which is a proxy for managent of antenatal complicatons were low and remained low in the study areas [33]. An integrated community and facility based interventions to increase utilization of ANC; timely recognition of antenatal complications and care seeking for managment of complications [6, 7] will be key strategies in reducing perinatal mortality in Bangladesh and similar settings. This will require creation of demand for these sevices including addressing barriers to access and improving avaialbility and quality of ANC and trained care for antenatal compliactions. Our study findings need to be replicated by future studies using objective measures of antepartum complications based on clinical examination and diagnosis.

## Abbreviations

APH: Antepartum hemorrhage
BP: Blood pressure
CHWs: Community health workers
CI: Confidence intervals
IMCI: Integrated management of childhood illness
IRR: Incidence rate ratios
PAF: Population attributable fraction
PIH: Pregnancy induced hypertension

## References

[1] Lawn JE, Blencowe H, Oza S, You D, Lee AC, Waiswa P, Lalli M, Bhutta Z, Barros AJ, Christian P (2014). Every newborn: progress, priorities, and potential beyond survival. Lancet (London, England).

[2] Lawn JE, Blencowe H, Waiswa P, Amouzou A, Mathers C, Hogan D, Flenady V, Frøen JF, Qureshi ZU, Calderwood C et al. Stillbirths: rates, risk factors, and acceleration towards 2030. Lancet. 2016. 10.1016/S0140-6736(15)00837-5 26794078

[3] Liu L, Johnson HL, Cousens S, Perin J, Scott S, Lawn JE, Rudan I, Campbell H, Cibulskis R, Li M (2000). Global, regional, and national causes of child mortality: an updated systematic analysis for 2010 with time trends since. Lancet (London, England).

[4] Lawn JE, Cousens S, Zupan J (2005). 4 million neonatal deaths: When? Where? Why?. Lancet (London, England).

[5] World Health Organization (2006). Neonatal and perinatal mortality : country, regional and global estimates.

[6] Owais A, Faruque ASG, Das SK, Ahmed S, Rahman S, Stein AD (2013). Maternal and antenatal risk factors for stillbirths and neonatal mortality in rural bangladesh: a case-control study. PLoS ONE.

[7] Vogel JP, Souza JP, Mori R, Morisaki N, Lumbiganon P, Laopaiboon M, Ortiz-Panozo E, Hernandez B, Perez-Cuevas R, Roy M (2014). Maternal complications and perinatal mortality: findings of the World Health Organization multicountry survey on maternal and newborn health. Bjog.

[8] Di MS, Say L, Lincetto O (2007). Risk factors for stillbirth in developing countries: a systematic review of the literature. Sex Transm Dis.

[9] McClure EM, Wright LL, Goldenberg RL, Goudar SS, Parida SN, Jehan I, Tshefu A, Chomba E, Althabe F, Garces A (2007). The global network: a prospective study of stillbirths in developing countries. Am J Obstet Gynecol.

[10] Macheku GS, Philemon RN, Oneko O, Mlay PS, Masenga G, Obure J, Mahande MJ (2015). Frequency, risk factors and feto-maternal outcomes of abruptio placentae in Northern Tanzania: a registry-based retrospective cohort study. BMC Pregnancy Childbirth.

[11] Adane AA, Ayele TA, Ararsa LG, Bitew BD, Zeleke BM (2014). Adverse birth outcomes among deliveries at Gondar University Hospital, Northwest Ethiopia. BMC Pregnancy Childbirth.

[12] Mamun AA, Padmadas SS, Khatun M (2006). Maternal health during pregnancy and perinatal mortality in Bangladesh: evidence from a large-scale community-based clinical trial. Paediatr Perinat Epidemiol.

[13] Yucesoy G, Ozkan S, Bodur H, Tan T, Caliskan E, Vural B, Corakci A (2005). Maternal and perinatal outcome in pregnancies complicated with hypertensive disorder of pregnancy: a seven year experience of a tertiary care center. Arch Gynecol Obstet.

[14] Sakornbut E, Leeman L, Fontaine P (2007). Late pregnancy bleeding. Am Fam Physician.

[15] Browne JL, Vissers KM, Antwi E, Srofenyoh EK, Van der Linden EL, Agyepong IA, Grobbee DE, Klipstein-Grobusch K (2015). Perinatal outcomes after hypertensive disorders in pregnancy in a low resource setting. Trop Med Int Health.

[16] Allanson ER, Muller M, Pattinson RC (2015). Causes of perinatal mortality and associated maternal complications in a South African province: challenges in predicting poor outcomes. BMC Pregnancy Childbirth.

[17] McClure EM, Nalubamba-Phiri M, Goldenberg RL (2006). Stillbirth in developing countries. Int J Gynaecol Obstet.

[18] Goldenberg RL, Thompson C (2003). The infectious origins of stillbirth. Am J Obstet Gynecol.

[19] RS G. The origins of stillbirth: infectious diseases. Semin Perinatol 2002, 26:75–78 10.1053/sper.2002.29839 11876570

[20] Vergnano S, Sharland M, Kazembe P, Mwansambo C, Health PT. Neonatal sepsis: an international perspective. Arch Dis Child Fetal Neonatal Ed. 2005;90:F220–4. 10.1136/adc.2002.022863 PMC1721871 15846011

[21] Sankar MJ, Agarwal R, Deorari AK, Paul VK. Sepsis in the newborn. Indian J Pediatr. 2008;75:261–6. 10.1007/s12098-008-0056-z 18376095

[22] Killewo J, Anwar I, Bashir I, Yunus M, Chakraborty J (2006). Perceived delay in healthcare-seeking for episodes of serious illness and its implications for safe motherhood interventions in rural Bangladesh. J Health Popul Nutr.

[23] Baqui AH, Williams E, El-Arifeen S, Applegate JA, Mannan I, Begum N, Rahman SM, Ahmed S, Black RE, Darmstadt GL (2016). Effect of community-based newborn care on cause-specific neonatal mortality in Sylhet district, Bangladesh: findings of a cluster-randomized controlled trial. J Perinatol.

[24] Baqui AH, El-Arifeen S, Darmstadt GL (2008). Effect of community-based newborn-care intervention package implemented through two service-delivery strategies in Sylhet district, Bangladesh: a cluster-randomised controlled trial. Lancet (London, England).

[25] Mitra SN, Al-Sabir A, Saha T, Sushil K (2001). Bangladesh demographic and health survey 199–2000.

[26] Darmstadt GL, Baqui AH, Choi Y (2009). Validation of community health workers’ assessment of neonatal illness in rural Bangladesh. Bull World Health Organ.

[27] Filmer D, Pritchett LH (2001). Estimating wealth effects without expenditure data--or tears: an application to educational enrollments in states of India. Demography.

[28] Rockhill B, Newman B, Weinberg C (1998). Use and misuse of population attributable fractions. Am J Public Health.

[29] Greenland S, Drescher K (1993). Maximum likelihood estimation of the attributable fraction from logistic models. Biometrics.

[30] National Institute of Population Research and Training (NIPORT), Mitra and Associates (MA), Macro International (2009). Bangladesh demographic and health survey, 2007.

[31] National Institute of Population Research and Training (NIPORT), Ministry of Health and Family Welfare, Mitra and Associates (MA), The DHS program, ICF International (2016). Bangladesh demographic and health survey 2014.

[32] Ellison GT, de Wet T, Matshidze KP, Cooper P (2000). The reliability and validity of self-reported reproductive history and obstetric morbidity amongst birth to ten mothers in Soweto. Curationis.

[33] National Institute of Population Research and Training (NIPORT) MaA, and ICF International (2015). Bangladesh demographic and health survey 2014: key indicators.

